# Extraperitoneal radical prostatectomy with the Senhance Surgical System robotic platform

**DOI:** 10.3325/cmj.2019.60.556

**Published:** 2019-12

**Authors:** Željko Kaštelan, Nikola Knežević, Tvrtko Hudolin, Tomislav Kuliš, Luka Penezić, Eleonora Goluža, Stefano Gidaro, Ante Ćorušić

**Affiliations:** 1 Department of Urology, University Hospital Centre Zagreb, Zagreb, Croatia *tkulis@gmail.com*; 2 University of Zagreb School of Medicine, Zagreb, Croatia; 3 Department of Anesthesiology, Reanimatology, and Intensive Medicine, University Hospital Centre Zagreb, Zagreb, Croatia; 4 Department of Medical, Oral and Biotechnological Sciences, University G. D'Annunzio, Chieti-Pescara, Italy; 5 Department of Gynecologic Oncology, Clinic for Gynecology and Obstetrics, University Hospital Centre Zagreb, Zagreb, Croatia

The introduction of Da Vinci (Intuitive Surgical, Inc., Sunnyvale, CA, USA) robotic surgical system for radical prostatectomy (RP) has significantly changed the way we perform prostate cancer surgery (1). In a relatively short time, Da Vinci has become the major surgical approach for RP in the USA (2). Furthermore, the number of European hospitals that use this system is constantly increasing. Until recently, Da Vinci was the only available robotic surgical system for robotic radical prostatectomy (RRP), but now several manufacturers already have such a system or are in the process of developing it (3). Recently, the Senhance Surgical System (TransEnterix, Inc., Morrisville, NC, USA) robotic platform for abdominal, gynecological, and urological surgical procedures has been introduced (4-8). This platform is feasible and safe, and it is relatively easy to learn to operate it (6,9,10). Here, we present our initial experience with this new system for radical prostatectomy, with the first technical description and a video of the operation.

In our hospital, we started to use the Senhance robotic platform in May 2019. We initially used it for suprarenal and renal surgery, after which we decided to move to RP. Our surgeons had reasonable experience with open and laparoscopic RP (LRP), which we consider to be an advantage in adopting Senhance RRP.

## The Senhance Surgical System

The Senhance system consists of an open surgical console, laparoscopic column, and up to four robotic arms. Its open-platform architecture allows the use of the existing laparoscopic equipment, thus reducing capital investments. The system features a 3D4K vision system, eye-tracking, ergonomic and comfortable surgical console, and haptic feedback. The instruments are reusable, available in various jaw patterns, and similar to laparoscopic instruments. Therefore, a surgeon experienced in laparoscopy can easily adapt and use this instrumentation. Furthermore, due to the durability and reusability of the instruments, and use of the usual reusable laparoscopic trocars, cost per operation is only slightly higher than that of traditional laparoscopy.

## Non-nerve sparing radical prostatectomy with the Senhance system

We decided to combine our previous laparoscopic approach with the approach used by our colleagues in Maxima Medical Center, Veldhoven, the Netherlands, who apply the Senhance system for RP. We describe our technique of non-nerve sparing extraperitoneal radical prostatectomy with the Senhance robotic surgical system step by step in Table 1 and Video 1.(supplementary video)

**Table 1:** Step by step surgical protocol description

Step	Surgical protocol description
1	Small infraumbilical incision (around 15 mm)
	Incising the fascia
	Blunt dissection with the finger to access the retropubic space
	Inflation of the balloon trocar in the preperitoneal space under camera control
	Insertion of the 10-mm camera trocar and closing of the skin incision with the two sutures
	Starting the insufflation
2	Placing the 10-mm and 5-mm trocars on each side under visual control
3	Docking the robot arms
	A middle trocar for the camera and lateral 5-mm trocars for working arms
	Left robotic arm for a bipolar instrument (Maryland Dissector ID Number 17)
	Right robotic arm for monopolar scissors (Curved Metzenbaum Scissors ID Number 67)
4	Two 10-mm trocars were used by the assistant surgeon for inserting a laparoscopic suction device, grasper, clip applicator, and/or advanced bipolar instrument
5	The surgeon moves to the surgical console
6	Removing the small amount of fatty tissue over the puboprostatic ligaments and dorsal veins
7	The incision of endopelvic fascia on both sides, from puboprostatic ligaments to the prostatic pedicle
8	Opening the anterior side of the bladder neck using bipolar and scissors
	Opening the posterior side using monopolar scissors
9	Identification of and cutting of *vas deferens* and seminal vesicles (with bipolar and scissors)
10	Developing the plane between the posterior side of the prostate and rectum
11	Lateral dissection of the prostate pedicles to the urethra (with bipolar and scissors)
	The assistant surgeon uses advanced bipolar or clip applicator under the supervision and guidance of the leading surgeon
12	Dividing the puboprostatic ligaments and dorsal vein complex (with bipolar and scissors)
13	Urethral sound is inserted to facilitate visualization
	Cutting the urethra
14	The prostate is mobilized, placed in an endobag, and pulled under the right 10-mm trocar
	Control of hemostasis
15	Change of robotic instruments
	Left arm for Maryland dissector (Maryland dissector, ID Number 69)
	Right arm for needle holder (Needle Holder Right No. 64)
16	Formation of vesicourethral anastomosis with barbed sutures
	The first suture is placed on the right side of the anastomosis at approximately 6 and 5 o’clock position
	Approximating the bladder neck and urethra to 1-2 cm
	The second suture is placed on the left side of anastomosis at approximately 7 and 8 o’clock position
	Completing the approximation of the bladder neck and urethra
	Placing the urethral catheter
	A continuous suture is placed at approximately 3 and 1 o’clock (right)
	A continuous suture is placed at approximately 9 and 11 o’clock (left)
	Tying both sutures
17	Checking the anastomosis for leaks with saline (200 mL)
18	Placing the drain
19	Removing the trocars and the prostate
20	Closing the wounds

**Figure 1:**
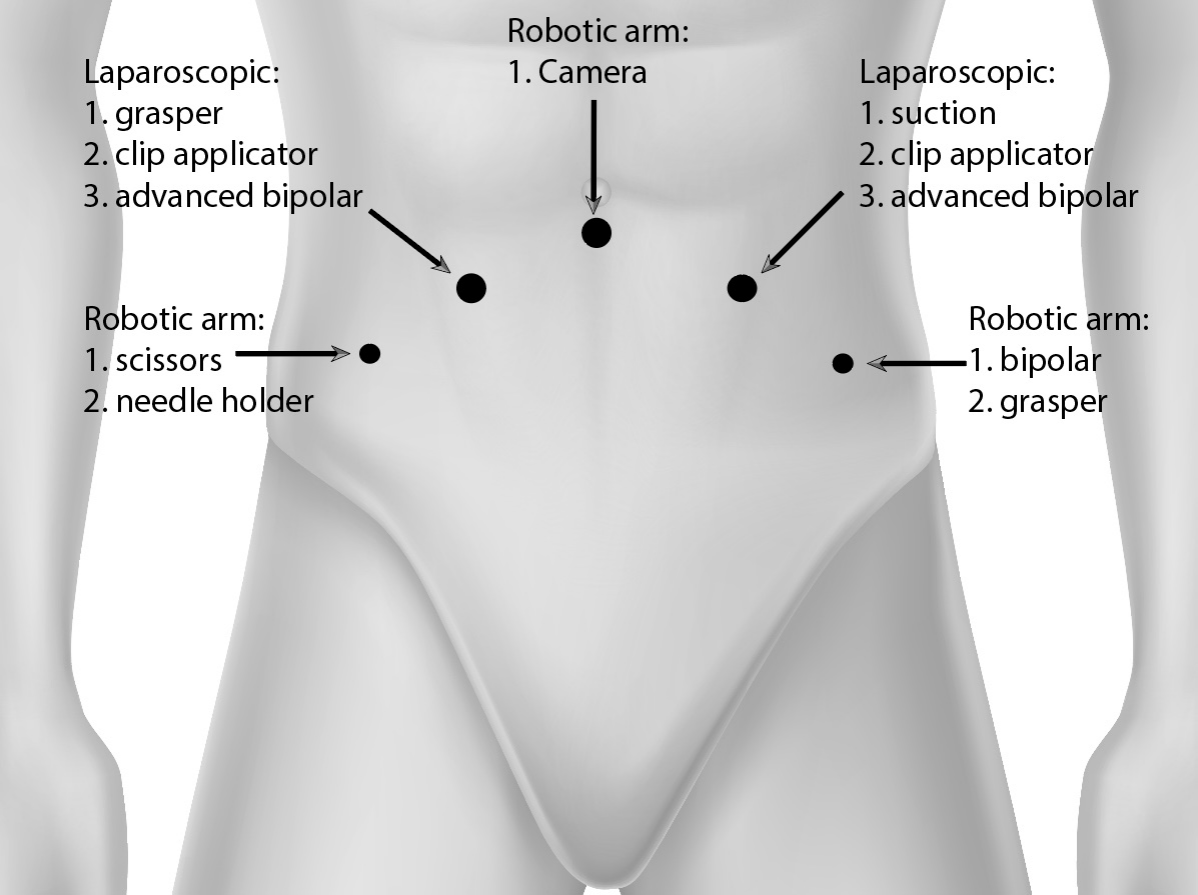
The position of the trocars is similar to the laparoscopic radical prostatectomy. The large circle represents 10-mm trocar and the small circle 5-mm trocar.

**Figure 2:**
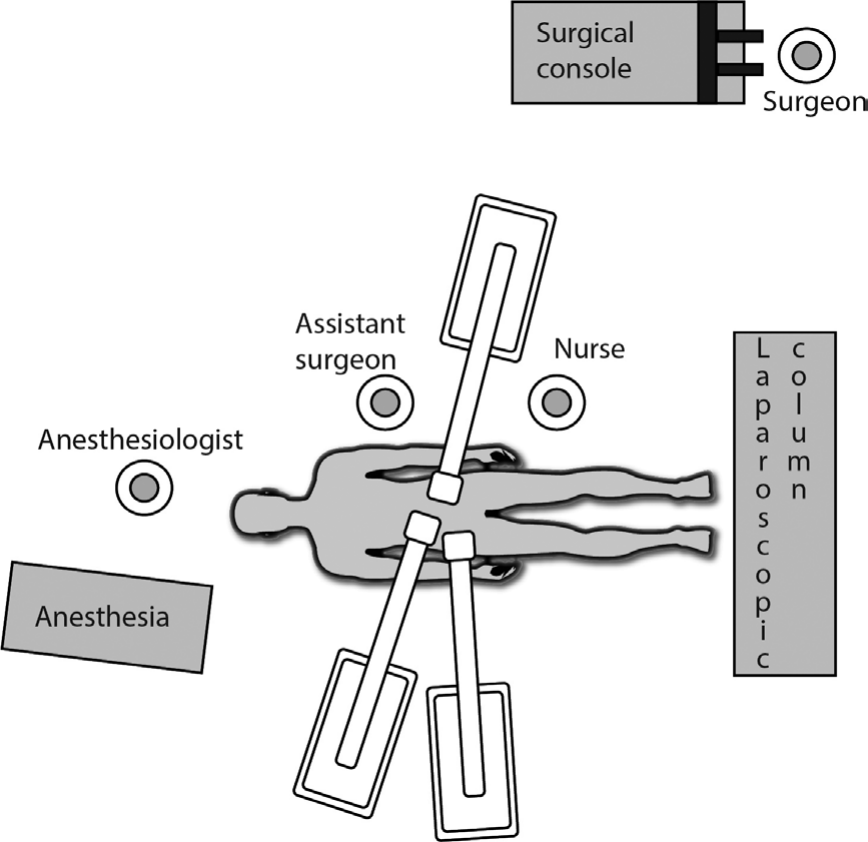
Position of the robotic arms and the assistant surgeon.

To insert the instruments, we used five openings-trocars, one in the midline for the camera, and two on both sides, as in the LRP (Figure 1). Three robotic arms were used for RRP and the other two instruments were under the control of an assistant surgeon (Figure 2). The drainage was removed after two days (when the secretion was <50 mL) and the catheter between seven and 10 days after surgery.

## Our experience

To learn any new technology or method, one must invest time and effort, not only from a surgical standpoint but from all other aspects of work in the operating room. Surgery is a team work and everybody in the team needs to be motivated and educated for their part of the job. When we started using the Senhance system, we spent up to one hour before surgery just to set up the system. It is mandatory to properly place the trocar/robotic instrument as robotic arms and instruments are relatively large and require sufficient space for ideal functioning. If they are placed too closely, they interfere with each other and the team, which can be a problem, and the system informs the surgeon that there is limited motion. We decided to separate the robotic arms as far as possible by placing them in the middle for the camera and laterally for the bipolar and scissors. Between them, we placed laparoscopic instruments operated by an assistant surgeon. The position of an assistant surgeon was on the left side, where only one robotic arm was located. Although this positioning was not ideal, it left him enough space and mobility to assist properly. Our previous laparoscopic experience indicates that the proper ability to hold/manipulate the prostate with a grasper is very important for easier and faster RP.

Between November and December 2019, we performed nine Senhance RRPs. The application of robotics was approved by our hospital’s Ethics Committee, and all patients gave written informed consent. The average age of patients was 64 years (range, 48-72). Operative time varied between 150 and 300 minutes, with an average of 217 minutes. Already the sixth operation lasted 150 minutes, showing a fast learning curve of the team. All steps were completed robotically, except in the first two cases, when the anastomosis was sutured laparoscopically.

## Conclusion

Here, we provide a technical description of our first experience with the Senhance system in radical prostatectomy. We believe that our experience can help other teams to more quickly learn how to use this method. Additionally, the learning curve can be particularly short in surgeons with previous LRP experience. The number of RRPs is increasing, and in the future this is likely to be the dominant type of surgery. We expect that new centers seeking lower maintenance and procedure costs could benefit from this system. Importantly, the conversion of RRP to LRP can be done simply, quickly, and safely, just by removing the robotic and by placing a laparoscopic instrument. Also, this type of RRP allows the use of an extraperitoneal approach, with which urologists are familiar and which has a benefit of not entering the peritoneal cavity. The use of a cockpit demands some time for adaptation, but with proper education and motivation, it provides benefits for the surgeon that compensate for the effort invested in the learning process.

The main disadvantage of the technique is additional time required to dock robot arms. However, as the team gets more experienced this can take less than 10 minutes. Furthermore, the Senhance system has a relatively large footprint, which reduces the working space for the assistant surgeon and the fourth robot arm.

The future will tell what role the Senhance system will play in RP, but, in our opinion, this robotic platform has some very important benefits, such as better visualization-magnification, 3D vision, eye-tracking technology, haptic feedback, and comfortable sitting position. Moreover, the instruments are reusable and the cost of maintaining and operating the platform is significantly lower compared with the Da Vinci system.
